# Differences in 5-Aminolevulinic Acid-Induced Hemodynamic Changes between Patients Undergoing Neurosurgery and Urological Surgery

**DOI:** 10.31662/jmaj.2021-0092

**Published:** 2021-09-27

**Authors:** Tohru Shiratori, Kunihisa Hotta, Masaaki Satoh, Atsushi Sato, Takayuki Kamigaito, Chiaki Kiuchi, Ryusuke Tanaka

**Affiliations:** 1 Department of Anesthesiology, Ina Central Hospital, Nagano, Japan; 2 Department of Anesthesiology and Critical Care Medicine, Jichi Medical University, Tochigi, Japan; 3 Department of Neurosurgery, Ina Central Hospital, Nagano, Japan; 4 Department of Urology, Ina Central Hospital, Nagano, Japan

**Keywords:** Aminolevulinic acid, glioma, urinary bladder neoplasms, hemodynamics, blood pressure, heart rate, hypotension, photodynamic therapy

## Abstract

**Introduction:**

Oral 5-aminolevulinic acid (5-ALA) is often used for photodynamic diagnosis-assisted glioma or bladder tumor surgery. 5-ALA affects blood pressure (BP). In fact, hypotension is a well-known adverse effect of 5-ALA in urology. However, information regarding 5-ALA-induced hemodynamic changes in neurosurgery remains limited. Furthermore, the duration of hypotension and how 5-ALA affects the heart rate (HR) are yet to be determined. Thus, in this study, we aimed to elucidate 5-ALA-induced perioperative hemodynamic changes in neurosurgery and urological surgery by examining real-world data.

**Methods:**

Consecutive patients who underwent neurosurgery (neurosurgery patients; 5-ALA-pretreated vs. non-pretreated [17 vs. 16], from January 2014 to March 2021) and urological surgery (urological surgery patients; 5-ALA-pretreated vs. non-pretreated [26 vs. 101], from August 2018 to September 2020) were enrolled. Differences in hemodynamics were evaluated using the linear mixed model. BP and HR in 5-ALA-pretreated patients were compared with those in non-pretreated patients. Differences in 5-ALA-induced preoperative BP changes were compared between the neurosurgery patients and urological surgery patients.

**Results:**

5-ALA scarcely affected the hemodynamics in neurosurgery patients, whereas 5-ALA-induced hemodynamic changes were clearly observed in urological surgery patients. Hemodynamic parameters were found to be not significantly different between 5-ALA-pretreated and non-pretreated neurosurgery patients. The preoperative, intraoperative, and postoperative BP in 5-ALA-pretreated urological surgery patients were significantly lower than those in the non-pretreated patients. Preoperatively, two 5-ALA-pretreated urological surgery patients had severe postural hypotension (systolic BP <50 mmHg), and one of them did not continue with the surgery because of prolonged severe hypotension. The BP in 5-ALA-pretreated urological surgery patients tended to be persistently lower for 9 h after 5-ALA pretreatment. The preoperative and postoperative HR values were higher in 5-ALA-pretreated urological surgery patients. Cumulative incidences of BP reduction and HR elevation were significantly higher in 5-ALA-pretreated urological surgery patients. The preoperative BP reduction in 5-ALA-pretreated urological surgery patients was significantly larger than that in neurosurgery patients.

**Conclusions:**

5-ALA-induced hemodynamics may differ between neurosurgery patients and urological surgery patients. 5-ALA may affect BP for at least 9 h.

## Introduction

5-Aminolevulinic acid (5-ALA) is a precursor of protoporphyrin IX, which is known to have high tumor selectivity and photoactivity. Photodynamic diagnosis (PDD) using 5-ALA has high sensitivity for tumor cell detection. Therefore, 5-ALA-mediated photodynamic therapy is a promising treatment for various cancers ^(1)^.

5-ALA was first used in neurosurgeries ^(2)^. Although hypotension is a 5-ALA-related adverse event ^(3), (4), (5), (6)^, it was considered insignificant in PDD-assisted neurosurgical trials ^(2), (7), (8), (9), (10)^. Therefore, hemodynamic changes following 5-ALA pretreatment in patients who underwent glioma surgery have been scarcely reported in neurosurgery. The same 5-ALA dosage in the preceding neurosurgical trials was used in subsequent urological clinical practices. After the clinical launch of 5-ALA in PDD-assisted urological surgery, 5-ALA-induced hypotension was observed to have frequently developed and was exacerbated at times ^(11)^. In severe cases, 5-ALA-induced hemodynamic changes occurred preoperatively and postoperatively persisted^(12), (13), (14)^. Reportedly, the real-world incidence of 5-ALA-induced hypotension was high in patients who underwent PDD-assisted urological surgery ^(15), (16), (17), (18), (19)^. However, the cause of 5-ALA-induced hypotension remains unknown. It is unclear how long hypotension lasts and how 5-ALA affects the heart rate (HR). Consequently, 5-ALA is used with caution in patients undergoing PDD-assisted urological surgery.

Chung et al. indicated the association between 5-ALA-induced hypotension and antihypertensive agents in patients who underwent neurosurgery (neurosurgery patients) ^(5)^. However, the absence of a control group and appropriate statistical analysis made their results inconclusive. Although some antecedent articles describe 5-ALA-induced hemodynamics in urological surgery and abdominal digestive surgery ^(11), (17), (20)^, no article details 5-ALA-induced hemodynamics in neurosurgery patients. Although it remains unclear why hypotension was unremarkable in the neurosurgical clinical trials, it is necessary to detail the 5-ALA-induced hemodynamics in neurosurgery patients. Moreover, no study has performed a comparison of 5-ALA-induced hemodynamics between neurosurgery patients and patients who underwent urological surgery (urological surgery patients). A comparison between these groups may yield useful insights regarding the management of 5-ALA-induced hypotension.

This observational study aimed to determine the 5-ALA-induced perioperative hemodynamics by analyzing the real-world data of neurosurgery patients and urological surgery patients.

## Materials and Methods

### Ethics

This single-center, non-blinded, non-randomized, retrospective observational study was approved by the ethics committee of Ina Central Hospital (approval number: 19-6, August 26, 2019), which waived the requirement for informed consent. This study was conducted according to the STROBE Statement.

### Survey period, population, and indications

#### Neurosurgery

We have retrospectively assessed consecutive patients who underwent neurosurgery for suspected glioma from January 2014 to March 2021. The indication for PDD-assisted surgery was determined based on device availability. General anesthesia with intravenous anesthetics was implemented for neurosurgery.

#### Urological surgery

We assessed consecutive patients who underwent transurethral resection of bladder tumor (TURBT) from August 2018 to September 2020. The exclusion criteria were as follows: concurrent major surgeries, TURBT duration >100 min, and postoperative hemorrhage requiring repeated TURBT. PDD-assisted surgeries indicated for non-muscle-invasive bladder cancer were determined based on device availability. Unless contraindicated or patients requested for general anesthesia, spinal anesthesia was implemented. General anesthesia was implemented with volatile anesthetics.

### Perioperative management of patients

Patients were requested to fast in the morning of surgery and were intravenously administered preoperative fluid without anesthetic premedication. Blood pressure (BP) was noninvasively measured before patients entered the operating room (OR). While the BP after anesthesia induction was measured invasively in neurosurgery patients, BP was measured noninvasively in urological surgery patients. During surgery, vasoactive agents were administered to maintain systolic BP (SBP) above 80 mmHg. Neurosurgery patients were postoperatively managed in the intensive care unit, whereas urological surgery patients were postoperatively managed in the general ward.

### Clinical data

We retrospectively extracted the following clinical data from the medical records: patient backgrounds, laboratory data, anesthesia data, operation data, BP values, and HR values.

Considering that patients took 5-ALA 20 mg/kg orally 3 h preoperatively, the time for recording BP and HR values was determined according to the time of patients’ entry to the OR. For patients who had no pretreatment before surgery, the assumed time that patients took a placebo was set at 3 h preoperatively. Six time zones (T) were designated for recording vital signs. The T in the early morning on the day of surgery was defined as T_BL_, the T before patients entered the OR was defined as T_2_ (2-2.5 h after the pretreatment), and the T immediately before anesthesia induction was designated as T_3_ (approximately 3 h after the pretreatment). For neurosurgery patients, intraoperative T were defined as T_4_ and T_6_ at approximately 4 and 6 h after the pretreatment (T_4_ from 3 to 5 h and T_6_ from 5 to 7 h after the pretreatment), respectively. For urological surgery patients, TURBT was performed approximately 4 h after the pretreatment. The intraoperative T was defined as T_4_, and the T from 5 to 7 h was defined as T_6_ in the post-TURBT period. For neurosurgery patients and urological surgery patients, the postoperative T from 8 to 10 h after the pretreatment was designated as T_9_. The minimum values of SBP (together with diastolic BP [DBP] and HR at that time) were recorded at the six T, which were defined as SBP_BL_ (DBP_BL_ and HR_BL_), SBP_2_ (DBP_2_ and HR_2_), SBP_3_ (DBP_3_ and HR_3_), SBP_4_ (DBP_4_ and HR_4_), SBP_6_ (DBP_6_ and HR_6_), and SBP_9_ (DBP_9_ and HR_9_). SBP_BL_, DBP_BL_, and HR_BL_ were considered baseline values.

The time courses of cumulative incidences of SBP reduction and HR elevation were compared via Kaplan-Meier curves. The chronological SBP and HR values measured in the ward preoperatively and postoperatively, excluding those in the OR, were also documented. Ward patients who had 20% SBP reduction and 20% HR elevation, both from baseline measurements, were counted.

### Primary and secondary analysis

The neurosurgery patients and urological surgery patients were categorized into 5-ALA-pretreated and non-pretreated patients for analysis. The main analysis was investigation into the hemodynamic change differences between the 5-ALA-pretreated and non-pretreated patients in neurosurgery and urological surgery, respectively. The secondary analysis included investigations regarding the difference in clinical data between the 5-ALA-pretreated neurosurgery patients and urological surgery patients, investigations into the rates of SBP change after pretreatment between the subgroups of the surgery-pretreatment category, and risk assessments of 5-ALA-induced intraoperative hypotension in urological surgery patients.

### Statistical analysis

All statistical analyses were performed using EZR on R commander (version 1.52) ^(21)^. P < 0.05 was considered statistically significant. Variables were expressed as median [interquartile range] or percentages. The ratio between two groups was compared using Fisher’s exact test. For continuous variables, two variables were compared using the Mann-Whitney U test.

As this retrospective survey was non-randomized, selection bias in PDD indication may have influenced the statistical results. Although the surgeon’s decision regarding the PDD-assisted surgery indication was unlikely to be an independent major confounder related to the patients’ hemodynamics, inverse probability weighting (IPW) was used for statistical adjustments to reduce possible selection biases derived from group-related confounding factors. Variables for the IPW included age, sex, body mass index, and comorbidity. The method of anesthesia in urological surgery was significantly different between the 5-ALA-pretreated and non-pretreated patients. As previously reported, general anesthesia may be associated with 5-ALA-induced hypotension ^(16), (17)^. Thus, the method of anesthesia was added as an IPW variable in the urological surgery patients. The interaction effects in the hemodynamic changes between the pretreatments and time courses were evaluated using the linear mixed model adjusted by IPW. Fluctuations in SBP, DBP, and HR in each group were analyzed using Friedman test with Wilcoxon signed rank test, followed by Bonferroni adjustment. The log-rank test was used to analyze cumulative incidences of 20% SBP reduction and 20% HR elevation, and the Cox proportional hazards model was used to calculate the hazard ratios, adjusted by IPW. The Kruskal-Wallis test was used for subgroup analysis to compare SBP change rates, followed by Bonferroni adjustment.

No statistical power calculation was conducted before this retrospective study. The data that support the findings of this study are available from the corresponding author upon reasonable request.

## Results

### Neurosurgery patients

The clinical data of 33 consecutive neurosurgery patients were collected. Based on the clinical courses, the patients were classified as 5-ALA-pretreated patients (n = 17) and non-pretreated patients (n = 16).

Table 1 shows a comparison of the clinical backgrounds between the 5-ALA-pretreated and non-pretreated neurosurgery patients. The 5-ALA-pretreated patients had longer operative time and larger blood loss than the non-pretreated patients.

**Table 1.** Comparison of Clinical Backgrounds between the 5-Aminolevulinic Acid-pretreated and Non-pretreated Patients Who Underwent Neurosurgery.

	5-ALA-pretreated	Non-pretreated	P-value
Number	17	16	
Age (yr)	64 [47–72]	71 [44–76]	0.871
Sex			0.303
Female	11 (64.7%)	7 (43.8%)	
Male	6 (35.3%)	9 (56.2%)	
BMI (kg/m^2^)	22.4 [20.6–24.6]	22.2 [20.5–24.1]	0.652
Tumor			
Recurrence	7 (41.2%)	3 (18.8%)	0.259
Pathology			1.000
Gliomas	15 (88.2%)	14 (87.5%)	
Lymphoma	0 (0.0%)	1 (6.2%)	
Metastatic tumor	2 (11.8%)	1 (6.2%)	
MRI volume (cm^3^)	37.6 [34.5–81.1]	24.5 [15.1–54.2]	0.292
KPS score	60 [50–60]	60 [50–80]	0.607
Comorbidity	7 (41.2%)	6 (37.5%)	1.000
Hypertension	5 (29.4%)	3 (18.8%)	0.688
Diabetes mellitus	0 (0.0%)	1 (6.2%)	0.485
Heart disease	0 (0.0%)	1 (6.2%)	0.485
Hyperlipidemia	3 (17.6%)	3 (18.8%)	1.000
CKD	0 (0.0%)	2 (12.5%)	0.227
Stroke	0 (0.0%)	0 (0.0%)	1.000
Laboratory data
TP (g/dL)	6.7 [6.4–7.0]	6.8 [6.7–7.2]	0.481
Alb (g/dL)	3.8 [3.5–4.2]	4.3 [3.9–4.5]	0.063
AST (IU/L)	16 [14–18]	23 [19–27]	0.008
ALT (IU/L)	15 [12–20]	18 [12–23]	0.329
Hb (g/dL)	12.6 [11.3–14.1]	13.7 [12.7–15.1]	0.528
Antihypertensives	5 (29.4%)	2 (12.5%)	0.398
RAS inhibitor	3 (17.6%)	1 (6.2%)	0.601
β-Blocker	0 (0.0%)	0 (0.0%)	N/A
Ca antagonist	2 (11.8%)	1 (6.2%)	1.000
Intraoperative
Operative time (min)	211 [161–228]	144 [126–151]	<0.001
SBP <80mmHg			
–T_4_	7 (41.2%)	6 (37.5%)	1.000
–T_6_	6 (35.3%)	6 (37.5%)	1.000
Vasoactives	16 (94.1%)	14 (87.5%)	0.601
Blood loss	130 [50–350]	45 [20–127]	0.024
Postoperative			
MV	15 (88.2%)	9 (56.2%)	0.057
Dopamine	2 (11.8%)	1 (6.2%)	1.000

Note: Data are presented as median [interquartile range] and numbers (proportion). Abbreviations: 5-ALA-pretreated, patients pretreated with 5-aminolevulinic acid; non-pretreated, patients who had no pretreatment before surgery; KPS, Karnofsky Performance Status; CKD, chronic kidney disease (estimated glomerular filtration rate <60 mL/min/1.73m^2^); RAS, renin-angiotensin system; N/A, not applicable; SBP, systolic blood pressure; T_4_ and T_6_, around 4 and 6 h after the pretreatment, respectively; MV, patients on mechanical ventilators at 9 h after pretreatments.

**Figure 1.**
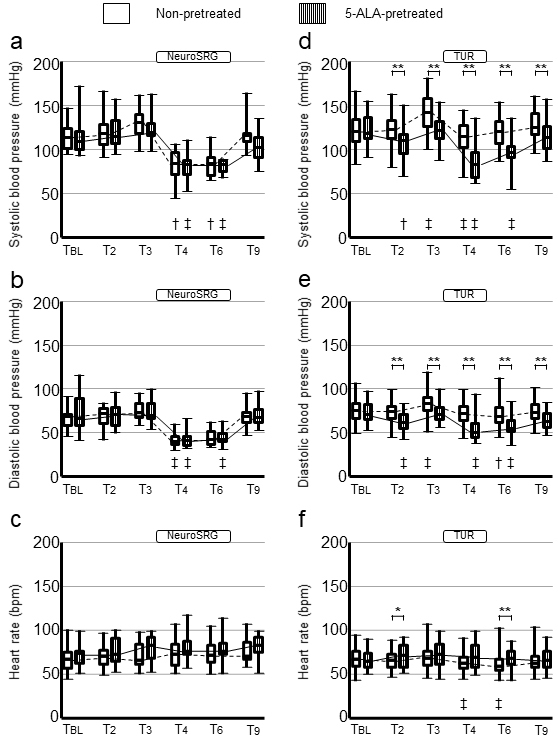
Differences of hemodynamics in patients who underwent neurosurgery and urological surgery between 5-aminolevulinic acid-pretreatment and non-pretreatment. a), b) and c): Systolic blood pressures, diastolic blood pressures, and heart rates during perioperative courses in patients who underwent neurosurgery. d), e) and f): those in patients who underwent urological surgery. Data are presented as medians [interquartile range]. ‡, P < 0.01; †, P < 0.05; comparison with baseline values using Wilcoxon signed rank test with Bonferroni adjustment following Friedman test. **, P < 0.01; *, P < 0.05; comparison between the 5-aminolevulinic acid-pretreated and non-pretreated patients using the Mann-Whitney U test. Abbreviations: Non-pretreated, patients who had no pretreatment before surgery; 5-ALA-pretreated, patients pretreated with 5-aminolevulinic acid; NeuroSRG, neurosurgery (brain tumor resection); TUR, urological surgery (transurethral resection of bladder tumor); T_BL_, on the early morning of the surgery day (before the pretreatments); T_2_, before patients entered the operating room (2-2.5 h after the pretreatment, preoperatively); T_3_, before anesthesia induction (3 h after the pretreatment, preoperatively); T_4_, amid operation (4 h after the pretreatment, intraoperatively); T_6_, amid operation in the neurosurgery (6 h after the pretreatment, intraoperatively) and after operation in the urological surgery (6 h after the pretreatment, postoperatively); T_9_, after operation (9 h after the pretreatment, postoperatively).

Figures 1a, b and c show changes in SBP, DBP, and HR, respectively, in the neurosurgery patients. The SBP, DBP, and HR values at T_BL_ were determined to be not significantly different between the 5-ALA-pretreated and non-pretreated patients. The interactions in SBP, DBP, and HR changes between pretreatments and time courses were nonsignificant (P = 0.169 [non-adjusted-P = 0.191]; P = 0.498 [non-adjusted-P = 0.502]; P = 0.657 [non-adjusted-P = 0.715], respectively). The BP and HR values at any designated time zones were nonsignificant between the 5-ALA-pretreated and non-pretreated patients.

**Figure 2.**
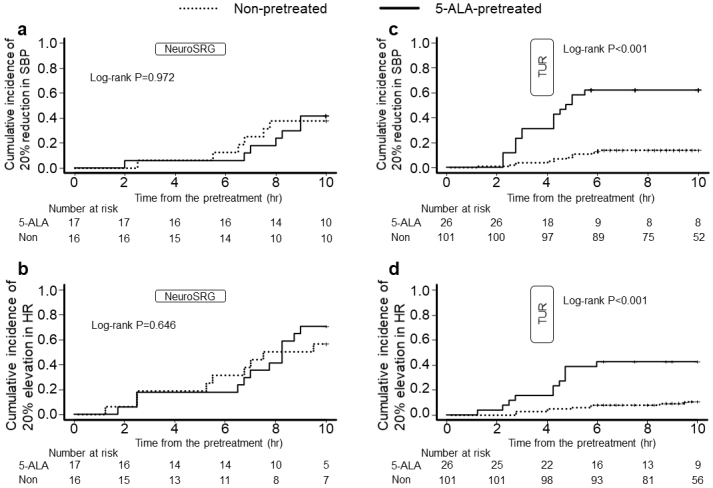
Kaplan-Meier curves of the cumulative incidence of systolic blood pressure reduction and heart rate elevation from baseline. a and c) Cumulative incidence of 20% reduction in systolic blood pressure from baseline in patients who underwent neurosurgery and urological surgery. b and d) Cumulative incidence of 20% elevation in heart rate from baseline in patients who underwent neurosurgery and urological surgery. Abbreviations: Non-pretreated (Non), patients who had no pretreatment before surgery; 5-ALA-pretreated (5-ALA), patients pretreated with 5-aminolevulinic acid; NeuroSRG, neurosurgery (brain tumor resection); TUR, urological surgery (transurethral resection of bladder tumor); SBP, systolic blood pressure; HR, heart rate.

The differences in Kaplan-Meier curves of the cumulative incidence of 20% SBP reduction and 20% HR elevation from the baseline were nonsignificant (log-rank test, P = 0.972 and 0.646, respectively; Figures 2a and b). The hazard ratios for SBP reduction and HR elevation in the 5-ALA-pretreated patients compared with those in the non-pretreated patients were 1.41 (95% confidence interval [CI], 0.47 to 4.26; P = 0.544) and 1.56 (95% CI, 0.64 to 3.80; P = 0.329), respectively.

### Urological surgery patients

Ten patients who underwent non-PDD-assisted TURBT were excluded because of surgery-related reasons. The clinical data of 127 consecutive patients who underwent TURBT were then collected. Based on the clinical courses, the patients were further classified as 5-ALA-pretreated patients (n = 26) and non-pretreated patients (n = 101).

Table 2 shows a comparison of the clinical backgrounds between the 5-ALA-pretreated and non-pretreated urological surgery patients. Four non-pretreated patients were transfused preoperatively due to severe anemia. The level of hemoglobin in the non-pretreated patients was significantly lower than that in the 5-ALA-pretreated patients. Among the 5-ALA-pretreated patients, the number of patients who were scheduled to undergo general anesthesia was larger than that among the non-pretreated patients. The 5-ALA-pretreated patients had a longer operative time and more frequent vasoactive administration than the non-pretreated patients. Two patients who received 5-ALA were determined to have severe postural hypotension (SBP <50 mmHg) preoperatively. One of them did not proceed with the surgery because of a persistent SBP of <80 mmHg. The SBP increased to >80 mmHg 7 h after 5-ALA administration but occasionally reduced to <70 mmHg at midnight despite continuous dopamine administration.

**Table 2.** Comparison of Clinical Backgrounds between the 5-Aminolevulinic Acid-pretreated and Non-pretreated Patients Who Underwent Urological Surgery.

	5-ALA-pretreated	Non-pretreated	P-value
Number	26	101	
Age (yr)	73 [63–77]	75 [64–79]	0.229
Sex			0.436
Female	4 (15.4%)	24 (23.8%)	
Male	22 (84.6%)	77 (76.2%)	
BMI (kg/m^2^)	23.0 [20.8–24.3]	23.1 [20.7–25.0]	0.986
Tumor			
Recurrency	13 (50.0%)	43 (42.6%)	0.515
Pathology			0.277
T_a_	11 (42.3%)	37 (36.6%)	
T_1_	7 (26.9%)	34 (33.7%)	
T_2_	0 (0.0%)	9 (8.9%)	
CIS	5 (19.2%)	8 (7.9%)	
Others	3 (11.5%)	13 (12.9%)	
Comorbidity	20 (76.9%)	75 (74.3%)	1.000
Hypertension	13 (50.0%)	54 (53.5%)	0.827
Diabetes mellitus	9 (34.6%)	19 (18.8%)	0.110
Heart disease	6 (23.1%)	11 (10.9%)	0.115
Hyperlipidemia	6 (23.1%)	28 (27.7%)	0.805
CKD	7 (26.9%)	32 (31.7%)	0.812
Stroke	1 (3.8%)	6 (5.9%)	1.000
Laboratory data
TP (g/dL)	6.8 [6.7–7.2]	7.0 [6.7–7.3]	0.217
Alb (g/dL)	4.2 [3.9–4.2]	4.1 [3.8–4.3]	0.629
AST (IU/L)	22 [19–27]	24 [21–29]	0.152
ALT (IU/L)	21 [14–28]	18 [14–25]	0.749
Hb (g/dL)	14.4 [13.9–15.2]	13.7 [12.8–14.7]	0.005
Preoperative BTF	0 (0.0%)	4 (4.0%)	0.581
Antihypertensives	13 (50.0%)	51 (50.5%)	1.000
RAS inhibitor	9 (34.6%)	28 (27.7%)	0.479
β-Blocker	1 (3.8%)	13 (12.9%)	0.298
Ca antagonist	7 (26.9%)	30 (29.7%)	1.000
Postural hypotension	2 (7.7%)	0 (0.0%)	0.041
Anesthesia plan			0.014
GA	7 (26.9%)	8 (7.9%)	
SA	19 (73.1%)	93 (92.1%)	
Intraoperative			
Operative time (min)	38 [26–55]	25 [16–33]	0.002
SBP <80mmHg	10 (40.0%)	2 (2.0%)	<0.001
Vasoactives	14 (53.8%)	3 (3.0%)	<0.001
Postoperative			
THP	19 (76.0%)	55 (54.5%)	0.069
Diclofenac	13 (52.0%)	33 (32.7%)	0.103
Hematuria			0.435
Non-visible	17 (70.8%)	58 (57.4%)	
Faint	3 (12.5%)	24 (23.8%)	
Gross	4 (16.7%)	19 (18.8%)	

Note: Data are presented as median [interquartile range] and numbers (proportion). Hematuria could not be evaluated in some patients owing to intravesical chemotherapy. Abbreviations: 5-ALA-pretreated, patients pretreated with 5-aminolevulinic acid; non-pretreated, patients who had no pretreatment before surgery; CKD, chronic kidney disease (estimated glomerular filtration rate <60 mL/min/1.73m^2^); BTF, blood transfusion; RAS, renin-angiotensin system; postural hypotension, preanesthetic severe postural hypotension (<50 mmHg); GA, general anesthesia; SA, spinal anesthesia; SBP, systolic blood pressure; THP, intravesical chemotherapy using pirarubicin immediately after surgery; hematuria, appearance of hematuria on the next early morning.

Figures 1d, e and f, show changes in SBP, DBP, and HR, respectively, in the urological surgery patients. The SBP, DBP, and HR values at T_BL_ were not significantly different between the 5-ALA-pretreated and non-pretreated patients. The interactions in SBP and DBP changes between pretreatments and time courses were significant (P = 0.009 [non-adjusted-P = 0.021] and P = 0.012 [non-adjusted-P = 0.004], respectively). The interaction in HR changes was nonsignificant between the 5-ALA-pretreated and non-pretreated patients (P = 0.172 [non-adjusted-P = 0.513]). The BP values in the 5-ALA-pretreated patients were significantly lower than those in the non-pretreated patients at T_2_, T_3_, T_4_, T_6_, and T_9_. The HR values in the 5-ALA-pretreated patients at T_2_ and T_6_ were significantly higher than those in the non-pretreated patients.

The differences in Kaplan-Meier curves of the cumulative incidence of 20% SBP reduction and 20% HR elevation from the baseline were determined to be significant (log-rank test, P < 0.001 and <0.001, respectively; Figures 2c and d). The hazard ratios for SBP reduction and HR elevation in the 5-ALA-pretreated patients compared with those in the non-pretreated patients were 7.45 (95% CI, 3.71 to 14.96; P < 0.001) and 5.98 (95% CI, 2.47 to 14.48; P < 0.001), respectively.

#### Differences in clinical backgrounds between 5-ALA-pretreated neurosurgery patients and urological surgery patients

Table 3 shows the difference in clinical backgrounds between the 5-ALA-pretreated neurosurgery patients and urological surgery patients. The 5-ALA-pretreated neurosurgery patients were determined to have higher proportion of female patients and lower prevalence of comorbidities than the urological surgery patients. Although the brain tumor volume was 37.6 [34.5-81.1] cm^3^, most urological surgery patients had superficial bladder tumors. The neurosurgery patients had lower levels of albumin, hemoglobin, and Karnofsky Performance Status.

**Table 3.** Comparison of Clinical Backgrounds of 5-Aminolevulinic Acid-pretreated Patients Who Underwent Neurosurgery and Urological Surgery.

	NeuroSRG	Urological-SRG	P-value
Number	17	26	
Age	64 [47–72]	73 [63–77]	0.098
Sex			0.002
Female	11 (64.7%)	4 (15.4%)	
Male	6 (35.3%)	22 (84.6%)	
BMI (kg/m^2^)	22.4 [20.6–24.6]	23.0 [20.8–24.3]	0.759
KPS score	60 [50–60]	100 [100–100]	<0.001
Tumor			
Recurrence	7 (41.2%)	13 (50.0%)	0.756
Tumor size			N/A
MRI volume (cm^3^)	37.6 [34.5–81.1]	N/A	
T (TNM classification)	N/A		
T_a_		11 (42.3%)	
T_1_		7 (26.9%)	
T_2_		0 (0.0%)	
CIS		5 (19.2%)	
Others		3 (11.5%)	
Comorbidity	7 (41.2%)	20 (76.9%)	0.026
Hypertension	5 (29.4%)	13 (50.0%)	0.219
Diabetes mellitus	0 (0.0%)	9 (34.6%)	0.007
Heart disease	0 (0.0%)	6 (23.1%)	0.066
Hyperlipidemia	3 (17.6%)	6 (23.1%)	1.000
CKD	0 (0.0%)	7 (26.9%)	0.031
Stroke	0 (0.0%)	1 (3.8%)	1.000
Antihypertensives	5 (29.4%)	13 (50.0%)	0.219
RAS inhibitor	3 (17.6%)	9 (34.6%)	0.306
β-Blocker	0 (0.0%)	1 (3.8%)	1.000
Ca antagonist	2 (11.8%)	7 (26.9%)	0.281
Laboratory data			
Alb (g/dL)	3.8 [3.5–4.2]	4.2 [3.9–4.2]	0.021
Hb (g/dL)	12.6 [11.3–14.1]	14.4 [13.9–15.2]	0.001
SBP <80mmHg (T_4_)	7 (41.2%)	10 (40.0%)	1.000

Note: Data are presented as median [interquartile range] and numbers (proportion). Abbreviations: NeuroSRG, patients who underwent neurosurgery; urological-SRG, patients who underwent urological surgery; KPS, Karnofsky Performance Status; CKD, chronic kidney disease (estimated glomerular filtration rate <60mL/min/1.73m^2^); RAS, renin-angiotensin system; SBP, systolic blood pressure; T_4_, around 4 h after the pretreatment (intraoperatively); N/A, not available.

#### Differences in SBP change rates between subgroups (surgery-pretreatment category)

**Figure 3.**
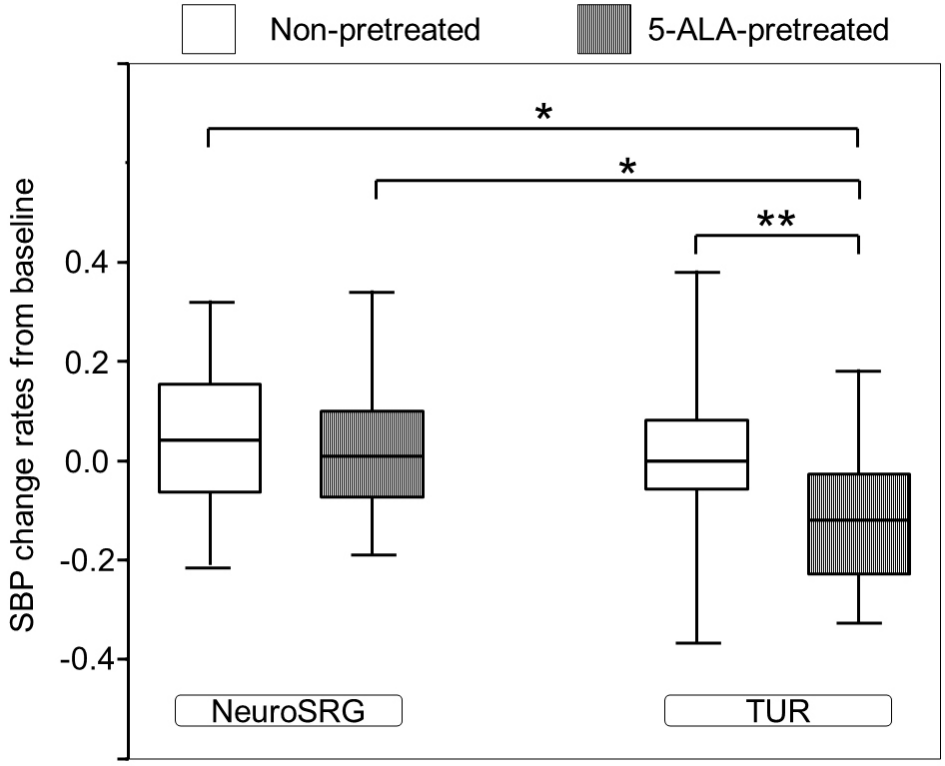
5-Aminolevulinic acid-induced changes in systolic blood pressure from baseline values before patients entered the operating room. Data are presented as medians [interquartile range]. **, P < 0.01; *, P < 0.05. Abbreviations: Non-pretreated, patients who had no pretreatment before surgery; 5-ALA-pretreated, patients pretreated with 5-aminolevulinic acid; SBP, systolic blood pressure; NeuroSRG, patients who underwent neurosurgery; TUR, patients who underwent urological surgery.

Of the designated T, T_2_ was considered to be less confounding because T_2_ was before anesthesia and surgery. Figure 3 shows the significant difference in SBP change rates at T_2_ from baseline values between subgroups classified by the surgery-pretreatment category (P < 0.001). The 5-ALA-pretreated patients in the subgroup of urological surgery exclusively had the largest SBP reduction. The SBP change rates in the 5-ALA-pretreated urological surgery patients were found to be larger than those in the 5-ALA-pretreated neurosurgery patients (P = 0.018), non-pretreated urological surgery patients (P < 0.001), and non-pretreated neurosurgery patients (P = 0.016). The SBP change rates in the non-pretreated neurosurgery patients did not differ significantly from those in the non-pretreated urological surgery patients (P = 1.000).

#### Relationships between intraoperative hypotension and clinical factors in 5-ALA-pretreated urological surgery patients

Table 4 shows the relationship between intraoperative hypotension and clinical factors. The correlation between intraoperative hypotension (SBP <80mmHg) and the use of antihypertensives was deemed significant (P = 0.015). Although general anesthesia may be a risk factor for 5-ALA-induced intraoperative hypotension ^(16), (17)^, the relation between intraoperative hypotension and anesthesia method was not significant in this study (P = 0.075).

**Table 4.** Relationships between 5-Aminolevulinic Acid-induced Intraoperative Hypotension and Clinical Backgrounds in Urological Surgery Patients.

	SBP < 80mmHg	SBP ≥80mmHg	P-value
Number	10	15	
Age (yr) ≥80	1 (10.0%)	2 (13.3%)	1.000
Sex			0.626
Female	1 (10.0%)	3 (20.0%)	
Male	9 (90.0%)	12 (80.0%)	
BMI (kg/m^2^) ≥25	3 (30.0%)	2 (13.3%)	0.358
Anesthesia			0.075
GA	5 (50.0%)	2 (13.3%)	
SA	5 (50.0%)	13 (86.7%)	
Comorbidity	8 (80.0%)	11 (73.3%)	1.000
Hypertension	8 (80.0%)	4 (26.7%)	0.015
Diabetes mellitus	4 (40.0%)	5 (33.3%)	1.000
Heart disease	3 (30.0%)	3 (20.0%)	0.653
Hyperlipidemia	3 (30.0%)	3 (20.0%)	0.653
CKD	3 (30.0%)	4 (26.7%)	1.000
Stroke	0 (0.0%)	1 (6.7%)	1.000
Antihypertensives	8 (80.0%)	4 (26.7%)	0.015
RAS inhibitor	4 (40.0%)	4 (26.7%)	0.667
Ca antagonist	3 (30.0%)	4 (26.7%)	1.000
β-Blocker	1 (10.0%)	0 (0.0%)	0.400
Pathology			0.353
T_a_	3 (30.0%)	8 (53.3%)	
T_1_	3 (30.0%)	4 (26.7%)	
CIS	2 (20.0%)	3 (20.0%)	
Others	2 (20.0%)	0 (0.0%)	

Note: Data are presented as numbers (proportion). The number of 5-aminolevulinic acid-pretreated patients for analysis was 25, because 1 patient did not proceed with the surgery due to a persistent SBP of <80 mmHg. Abbreviations: SBP <80mmHg, patients who had intraoperative hypotension below 80 mmHg; SBP ≥80mmHg, patients who had intraoperative minimum systolic blood pressure above 80 mmHg; GA, general anesthesia; SA, spinal anesthesia; CKD, chronic kidney disease (estimated glomerular filtration rate <60mL/min/1.73m^2^); RAS, renin-angiotensin system.

## Discussion

First, as the main finding in this retrospective case-control observational study, perioperative hemodynamic changes in urological surgery were significantly different between the 5-ALA-pretreated and non-pretreated patients, whereas the perioperative hemodynamic changes in the 5-ALA-pretreated neurosurgery patients were likely to be similar to those in the non-pretreated neurosurgery patients. Cumulative incidences on SBP reduction and HR elevation were determined to be significant between the 5-ALA-pretreated and non-pretreated urological surgery patients. Furthermore, the 5-ALA-induced SBP change rates from baseline in the urological surgery patients were larger than those in the neurosurgery patients. Second, the hemodynamic changes in the 5-ALA-pretreated urological surgery patients developed before patients entered the OR and lasted at least 9 h after 5-ALA pretreatment. Third, the use of antihypertensives was a significant factor for intraoperative hypotension in the 5-ALA-pretreated urological surgery patients. These results provide useful clinical information regarding 5-ALA-induced hemodynamic changes for safe perioperative management.

First, 5-ALA was able to decrease BP in the urological surgery patients preoperatively, operatively, and postoperatively, whereas it seemed to scarcely affect BP in the neurosurgery patients (Figure 1). Kaplan-Meier curves distinctly figure the significant difference in cumulative incidences of 5-ALA-induced hemodynamic changes in the urological surgery patients (Figure 2). The subgroup analysis between surgery-pretreatment categories has exclusively showed the significant difference of SBP change rates in the 5-ALA-pretreated urological surgery patients (Figure 3). This may indicate that the effect of 5-ALA on hemodynamics was different between the neurosurgery patients and urological surgery patients. In our survey conducted in the urological surgery patients, 5-ALA-induced hypotension was clearly observed, which closely corroborates previous findings ^(17), (19)^. Figures 1a, b and c show that the hemodynamic changes in the 5-ALA-pretreated neurosurgery patients were similar to those in the non-pretreated patients. The number of neurosurgery patients was limited. Moreover, the neurosurgery patients had factors that could substantially influence perioperative hemodynamics, including surgical procedures, anesthetic managements, and intensive care. Therefore, the same method used for hemodynamic investigations in the urological surgery patients may reduce the statistical power to detect 5-ALA-induced hemodynamic changes in the neurosurgery patients. General anesthesia is a factor influencing 5-ALA-induced intraoperative hypotension ^(16), (17)^. The proportion of 5-ALA-pretreated urological surgery patients who underwent the procedure under general anesthesia was larger than that of non-pretreated urological surgery patients in this study (Table 2). The difference of anesthesia type in the urological surgery patients may have influenced the frequency of 5-ALA-induced hypotension in the middle of urological surgery. However, adjustment of anesthesia type was implemented for analysis in the urological surgery patients. Furthermore, 5-ALA-induced BP reduction was significant even in the period unrelated to anesthesia (Figure 1). Thus, the difference in the type of anesthesia had little influence on interpretations of the analysis for urological surgery patients. Consequently, the urological surgery patients were determined to be vulnerable to 5-ALA-induced hemodynamic effects, whereas the neurosurgery patients were resilient to 5-ALA-induced hemodynamic effects.

The patients’ clinical backgrounds significantly differed between the 5-ALA-pretreated neurosurgery patients and urological surgery patients in terms of sex, comorbidity, and tumor size (Table 3). The neurosurgery patients had more female patients and fewer comorbidities than the urological surgery patients. A previous research lists female sex as a risk factor for 5-ALA-induced hypotension ^(16)^. Severe 5-ALA-induced hypotension developed in a patient without treatment for comorbidities ^(13)^. A high proportion of women and low rate of comorbidities in neurosurgery patients are unlikely to be independent major factors for the absence of 5-ALA effects.

The neurosurgery patients may have had pathologically larger tumors than the urological surgery patients, although a statistical comparison of tumor size could not be performed due to differences in tumor type. Although a preoperative volumetry for brain tumor in the neurosurgery patients showed that the median tumor volume was 37.6 cm^3^, most urological surgery patients had superficial bladder tumors. According to a previous report, the bladder tumor volumetry before radiotherapy recorded the mean volume as 29 cm^3^ in patients with invasive bladder tumors staged in T3 and T4 (Invasive-BT) ^(22)^. The superficial bladder tumor volume is considered to be much smaller than Invasive-BT. Although no study has compared tumor volumes between brain and bladder tumors, the neurosurgery patients were inferred to have a larger tumor volume than the urological surgery patients. Considering that 5-ALA is highly selective to accumulate in tumors, the amount of 5-ALA freely distributed in non-tumor tissues is inferred to be larger in urological surgery patients compared in neurosurgery patients, resulting in an excessive 5-ALA dose for urological surgery patients. Previous reports have reported the dose-dependent hemodynamic effects of 5-ALA ^(15), (18)^. There is no report on the relationship between tumor size and pharmacokinetics of 5-ALA; thus, it is necessary to investigate the minimum dose necessary for PDD-assisted urological surgery. Furthermore, the neurosurgery patients had lower Karnofsky Performance Status scores and lower levels of hemoglobin and albumin than the urological surgery patients. The neurosurgery patients were considered to have a lower nutrition status than the urological surgery patients. Further investigation is required to determine whether 5-ALA-induced hemodynamic changes differ in magnitude based on patients’ clinical backgrounds.

Moreover, in the urological surgery patients, 5-ALA-induced BP reduction began before patients entered the OR and lasted approximately 9 h after the pretreatment (Figures 1d, 1e and 2c). Thus, anesthesia induction was in the middle of 5-ALA-induced hypotension, and 5-ALA had prolonged effects on hemodynamics. Although a previous study reported that the effect of 5-ALA lasted 2 h after TURBT ^(19)^, few studies have examined the duration of the effects of 5-ALA on hemodynamics. In our postoperative management of the urological surgery patients, BP was not practically measured at night if the general condition was stable. Thus, we could not observe hemodynamic changes over 10 h after 5-ALA administration in most cases. Prolonged 5-ALA-induced vasoplegic shock has also been previously reported ^(12), (14), (15)^. Therefore, it is necessary to confirm whether the effect of 5-ALA is prolonged for more than 10 h after administration. Nevertheless, the lower BP trends in the 5-ALA-pretreated patients were restored over time. Although the 5-ALA-induced hemodynamic changes could extend to more than 10 h ^(15)^, the long-term effects may be vague in most patients. Herman et al. reported that systemic vascular resistance (SVR) reduction was noted within 4 h after 5-ALA administration ^(20)^. The BP reduction in the 5-ALA-pretreated urological surgery patients could have resulted from the 5-ALA-induced reduction in SVR. Changes in HR may have resulted from baroreceptor reflexes against 5-ALA-induced hypotension. Although the 5-ALA-induced SVR reduction is suspected to involve long-term hemodynamic changes, it remains unknown whether 5-ALA-induced postoperative hemodynamic changes solely depend on the SVR reduction. While the 5-ALA level begins to decrease 2 h after administration, a higher level of protoporphyrin IX is detected until ≥ 10 h ^(23), (24)^. It is inferred that 5-ALA long-lasting hemodynamics is associated with subsequent metabolic pathways rather than the direct effect of 5-ALA.

Furthermore, the subgroup analysis revealed a significantly higher proportion of antihypertensive use in the 5-ALA-pretreated urological surgery patients who had intraoperative hypotension (Table 4). According to previous reports on 5-ALA-induced hypotension, risk factors include age ^(18)^, cardiovascular disease ^(5), (15)^, use of antihypertensives ^(5), (17)^, general anesthesia ^(16), (17)^, and renal dysfunction ^(18)^. Risk factors for 5-ALA-induced hypotension apparently vary according to study methods, which is attributed to the fact that previous studies had different outcomes in the assessment of 5-ALA-induced hypotension. Among the risk factors described in previous studies, antihypertensive use was confirmed as a factor for 5-ALA-induced intraoperative hypotension, despite the limited number of cases in this study. Antihypertensives may synergically act on 5-ALA-induced intraoperative hypotension.

Due to its single-center, non-blinded, non-randomized, and retrospective design, this study has some limitations when considering the possible bias in the results. Although the study may have had some selection bias, neither the patients’ backgrounds nor the laboratory results influenced the surgeons’ decisions regarding the indication for PDD-assisted surgery. Although the hypotensive effect of 5-ALA differed between neurosurgery patients and urological surgery patients, prospective studies are necessary to confirm whether the difference depends on different tumors or different patient backgrounds. Studies of 5-ALA pharmacodynamics are also insufficient. The real-world clinical data have not been analyzed in detail. Pharmacological studies of 5-ALA in the absence of surgery and anesthesia may be required to accurately assess the effects of 5-ALA.

In conclusion, this retrospective study detailed the 5-ALA-induced hemodynamic changes in neurosurgery patients and urological surgery patients. The effects of 5-ALA on hemodynamics were exclusively observed in urological surgery patients compared with those in neurosurgery patients. After 5-ALA administration, BP was noted to decrease, which lasted for 9 h in urological surgery patients. It may be appropriate to investigate the 5-ALA-induced hemodynamic changes in neurosurgery patients separately from those in urological surgery patients.

## Article Information

### Conflicts of Interest

None

### Acknowledgement

We gratefully acknowledge Toyomi Kamesaki (Division of Community and Family Medicine, Center for Community Medicine, Jichi Medical University) and Yoshinobu Kanda (Division of Hematology, Department of Medicine, Jichi Medical University). The authors also thank the Clinical Research Support Team Jichi (CRST) in Jichi Medical University for their advice. This study was not financially supported.

### Author Contributions

Concept and design: TS, KH, and MS; drafting the manuscript: TS; revising the manuscript: TS, KH, and MS; data collection: TS; statistical analysis: TS; interpretation of the data: TS, AS, TK, CK, and RT; neurosurgery: AS; urological surgery: TK. Authors have read and approved the final manuscript.

### Approval by Institutional Review Board (IRB)

Approval number: 19-6, August 26, 2019 (the ethics committee of Ina Central Hospital).
